# The American Dental Association

**Published:** 1891-09

**Authors:** 


### EDITORIAL.

### The American Dental Association.

The thirty-first annual session of this body met at Saratoga, August 5th, continuing four days. The meeting was well attended by the representative men of the profession throughout the country; perhaps not as large a number in attendance as there has sometines been heretofore, but we were agreeably surprised to find so many of the older members present. The work done in all respects by the meeting was quite equal to that of former years. That the method of work pursued at present and heretofore, is not wholly satisfactory was quite clearly indicated in the address of the president, together with the fact that a committee was appointed to take into consideration the suggestions made in the address, in reference to a change in the organization. This committee after due consideration recommended several radical changes which it is supposed, by many at least, will serve to make the work of the body more efficient, and free its legitimate work from much business embarrassment that has heretofore existed.

Five years ago the following standing resolution was adopted:

*Resolved*, "That any member of the dental profession who has been in reputable practice for a period of fifty years may be elected to permanent membership in this association without the payment of dues; and any member of this association who has been in practice for a like period shall have his dues remitted thereafter by presenting the fact to the treasurer of the association."

At that time one permanent member was elected in accordance with that resolution, viz., Dr. W. H. Dwinelle, of New York, who we are happy to know is still in active practice, though he has passed his fifty-second year of practice; he was also present at this meeting, seemingly hale and hearty as ever, and was ready to measure strength with any of the "boys." This year two other members came in for this honorable distinction of

Half Century Practitioners, viz: Dr. John B. Rich, of Washington City, and Dr. Corydon Palmer, of Warren, Ohio. Both of these men are known, and have an enviable reputation not only in this country, but throughout the world wherever dentistry is practiced. The man who can present a record of fifty years of reputable practice and service for his profession, is certainly entitled to more than ordinary honors. It is a grand thing to live fifty years in one continuous occupation, and a greater thing to have sustained and added to the honor and resources of that calling or profession throughout such a period.

One of the most interesting incidents of this meeting was brought about by Dr. Palmer, who has had prepared, by one of the finest artists in New York, a life-size portrait of the late Dr. W. H. Atkinson, which had been finely framed. The doctor asked the privilege of presenting a little matter to the association on his own account: this he did by placing upon the wall the portrait covered with canvass, and just at the moment when all who were most interested to know what was coming, the doctor unveiled the portrait with some very happy remarks appropriate for such an one as Dr. Atkinson, at the conclusion of which he presented the portrait to the association. The scene was a very striking one, and produced a hushed solemnity in the meeting.

A resolution of thanks was voted to Dr. Palmer which was most cordially given, and the present accepted. Dr. Palmer was made the custodian of the portrait until the society shall have a secure and fixed place for its deposit.

In regard to Dr. J. B. Rich, the other honorable member, his personal vigor, health and professional interest were a marvel to all present. He is as ever, in the active practice of his profession and seemingly fit for many years more of service.

An installment of a report of the proceedings of this meeting appears in the present number of the REGISTER; it will be completed in the next. The publication committee have the transactions in the hand of the printer, and doubtless it will be issued ere long.

